# Caractérisation moléculaire de souches algériennes de *Blastocysts* sp

**DOI:** 10.48327/mtsi.v2i1.2022.226

**Published:** 2022-03-02

**Authors:** Fatma BACHI, Fayçal ABIDAT, Yasmine GHAFFOR, Sarra BELLILI, Soumaya GOURA, Sid Ali BELMADANI

**Affiliations:** 1 Laboratoire de biologie parasitaire, Département de parasitologie, Institut Pasteur d’Algérie, Route du petit Staouéli, Dely-Brahim, Alger, Algérie; 2 Département de médecine, Faculté de médecine d’Alger, Université d’Alger, Algérie

**Keywords:** Génotypage, *Blastocystis* sp, Souches algériennes, Maghreb, Afrique du Nord, Genotyping, *Blastocystis* sp, Algerian strains, Maghreb, Northern Africa

## Abstract

**Introduction:**

*Blastocystis* sp. est un protozoaire colonisant le tube digestif de l’Homme et de nombreux animaux et il est à ce jour le parasite le plus fréquemment retrouvé dans les selles humaines. Dans certains pays en développement, sa prévalence dans les populations étudiées peut dépasser 50 %. Sur le plan morphologique, les isolats de *Blastocystis* sp. trouvés chez différents hôtes sont très similaires. Cependant, ces mêmes isolats présentent entre eux une très grande diversité génétique et pas moins de 17 sous-types (ou génotypes) ont déjà été identifiés à partir de données moléculaires. Des études de génotypage ont été réalisées dans de nombreux pays à travers le monde et en particulier dans certains pays du pourtour méditerranéen comme la France, l’Espagne, l’Italie, la Turquie ou l’Égypte. En revanche, très peu de données de génotypage sont disponibles en Algérie. À cet effet, nous avons réalisé la présente étude dont le but était d’identifier et de génotyper *Blastocystis* dans des échantillons de selles humaines et animales.

**Patients et méthodes:**

Mille huit cent soixante-neuf (1 869) prélèvements de selles appartenant à un personnel de cuisine dans le cadre du contrôle médical périodique, à des sujets pour la fourniture d’un certificat médical requis pour le traitement d’un dossier de visa et à des patients atteints de troubles gastro-intestinaux ont été examinés. À côté des selles humaines, des échantillons d’animaux, dont 10 volailles, 2 bovins et 2 murins ont été examinés. Toutes les selles ont été soumises à un examen microscopique direct complété par des techniques de concentration et une coloration de Ziehl Neelsen modifiée. La caractérisation moléculaire de 39 isolats humains et 14 d’origine animale a été réalisée par le séquençage et les séquences obtenues comparées à celles disponibles auprès de GenBank. Le séquençage n’a été contributif que pour 30 souches humaines et 9 souches animales.

**Résultats:**

Sur l’ensemble des échantillons humain examinés, 284 étaient positifs (15,19 %) avec une prévalence de 7,38 % pour le *Blastocystis.* Parmi les 30 souches ayant fait l’objet d’une caractérisation moléculaire, le ST3 était prédominant (15/30, 50 %) suivi de ST1 (10/30, 33,33 %) et en troisième position le ST2 (4/30, 13,33 %). Le ST4 n’a été identifié que chez un seul patient (1/30, 3,33 %). La corrélation entre le statut clinique et le sous-type de *Blastocystis* identifié a montré que le nombre de ST3 était élevé chez les sujets asymptomatiques (11/15, 73 %) comparativement aux sujets symptomatiques (4/15, 26,66 %), de même pour le sous-type ST1 (7/10, 70 % versus 3/10, 30 %). À l’inverse, le nombre de ST2 était plus élevé chez les sujets présentant des troubles gastro-intestinaux (3/4, 75 %). À côté des souches humaines nous avons génotypé 7 souches aviaires, 2 souches murines et 2 souches bovines. La caractérisation des souches aviaires a révélé 5 ST6 (71,42 %) et 2 ST7 (28, 57 %). Les souches murines et bovines sont identifiées en tant que ST7 et ST6 respectivement.

## Abstract (en)

**Introduction:**

*Blastocystis* sp. is a protozoan that colonizes the gastrointestinal tract of humans and many animals and is currently the most common parasite found in human stools. In some developing countries, its prevalence in study populations may exceed 50%. Morphologically, isolates of *Blastocystis* sp. found in different hosts are very similar. However, these same isolates show a very high genetic diversity between them and no less than 17 subtypes (or genotypes) have already been identified from molecular data. Genotyping studies have been carried out in many countries around the world and in particular in some Mediterranean countries such as France, spain, Italy, Turkey and Egypt. However, very little genotyping data is available in Algeria. To this end, we conducted the present study to identify and genotype *Blastocystis* in human and animal stool samples.

**Patients and methods:**

One thousand eight hundred and sixty-nine (1,869) stool samples from kitchen staff as part of the periodic medical check-up, from subjects for the provision of a medical certificate required for the processing of a visa file and from patients with gastrointestinal disorders were examined. In addition to human faeces, animal samples, including 10 poultry, 2 cattle and 2 murine animals were examined. All stools were subjected to direct microscopic examination supplemented by concentration techniques and modified Ziehl Neelsen staining. Molecular characterization of 39 human and 14 animal isolates was performed by sequencing and the resulting sequences compared with those available from GenBank. Sequencing was only contributory for 30 human and 9 animal strains.

**Results:**

Of all human samples examined 284 were positive (15.19%) with a prevalence of 7.38% for *Blastocystis.* Of the 30 strains that were molecularly characterized, ST3 was predominant (15/30, 50%) followed by ST1 (10/30, 33.33%) and in third place ST2 (4/30, 13.33%). ST4 was identified in only one patient (1/30, 3.33%). The correlation between clinical status and the subtype of Blastocystis identified showed that the number of ST3 was high in asymptomatic subjects (11/15, 73%) compared to symptomatic subjects (4/15, 26.66%), as well as for the ST1 subtype (7/10, 70% versus 3/10, 30%). Conversely, the number of ST2 was higher in subjects with gastrointestinal disorders (3/4, 75%). In addition to human strains, we genotyped 7 avian, 2 murine and 2 bovine strains. Characterization of the avian strains revealed 5 ST6 (71.42%) and 2 ST7 (28, 57%). The murine and bovine strains are identified as ST7 and ST6 respectively.

## Introduction

Considéré comme un protozoaire parasite émergent, *Blastocystis* sp. est retrouvé dans le tractus intestinal de l’Homme mais aussi de divers animaux. Cet eucaryote est actuellement le parasite le plus fréquemment rencontré dans les selles chez l’Homme, et présente une prévalence beaucoup plus élevée que d’autres protozoaires intestinaux tels que *Giardia, Entamoeba* ou *Cryptosporidium* [7].

Sa prévalence varie considérablement selon les régions géographiques. Elle est de 100 % dans les pays en développement et de 56 % dans les pays industrialisés, variant d’un pays à l’autre, voire d’une population à l’autre pour un même pays [15,26,31,33]. Cette différence peut s’expliquer par le manque d’hygiène, le contact avec certains animaux et la consommation d’eau ou de nourriture contaminée par des kystes, la voie oro-fécale étant considérée comme la voie de contamination privilégiée de *Blastocystis* sp. [16, 20, 22]. Les données de prévalence dépendent également de la méthode de diagnostic utilisée, en particulier la microscopie dont la sensibilité est moindre comparativement aux techniques moléculaires dont la PCR.

La pathogénie de *Blastocystis* sp. est cependant controversée, du fait que ce parasite peut être retrouvé chez des patients symptomatiques mais aussi chez des patients asymptomatiques [12, 31]. Néanmoins, de nombreuses études in vitro et in vivo ont montré que *Blastocystis* sp. est capable de sécréter certains facteurs de virulence, comme des protéases, pouvant conduire à une altération de l’épithélium intestinal de l’hôte [25].

Le tableau clinique de l’infection à *Blastocystis* sp. regroupe de nombreux symptômes non spécifiques tels que la diarrhée, les douleurs abdominales et des lésions d’urticaire [21, 32]. En outre, *Blastocystis* sp. a été récemment associé au côlon irritable (IBS) [24].

Au niveau moléculaire, le genre *Blastocystis* présente une grande diversité génétique [2]. Dix-sept sous-types (ST1 - ST17) ont été identifiés dont 9 (ST1 - ST9) ont été trouvés chez l’Homme avec une prévalence variable [3]. Dans la plupart des pays du monde, ST3 est globalement prédominant suivi de ST1 et ST2, alors que ST4 est rarement trouvé en dehors de l’Europe. Ces 9 ST humains sont également partagés par divers hôtes non humains, soulignant à la fois la faible spécificité d’hôte du parasite et son potentiel zoonotique [2].

Malgré l’impact potentiel de *Blastocystis* sp. en santé publique, peu de publications sont disponibles sur la prévalence et la distribution des ST du parasite dans certaines régions géographiques dont l’Algérie. C’est dans ce contexte que s’inscrit notre étude afin de connaître la prévalence du parasite dans une population de patients, d’acquérir des données moléculaires et d’évaluer la distribution des ST dans la population algérienne.

## Patients et Méthodes

Un total de 1 869 selles a été collecté dans le cadre de l’activité diagnostic du laboratoire de biologie parasitaire appartenant au département de parasitologie à l’Institut Pasteur d’Algérie durant une période de trois ans, de janvier 2016 à décembre 2018. Notre échantillonnage est représenté essentiellement par les selles du personnel de cuisine (chaîne d’hôtels) dans le cadre du contrôle médical périodique, des sujets pour l’acquisition d’un certificat médical requis pour le traitement d’un dossier de visa et des patients atteints de troubles gastro-intestinaux. Il est de ce fait à prédominance de sujets asymptomatiques.

Toutes les selles ont été soumises à un examen direct suivi d’une technique de concentration au formol-éther (technique de Ritchie modifiée) et de la coloration d’un frottis selon la technique de Ziehl Neelsen modifiée pour la recherche de *Cryptosporidium* sp.

En plus des selles humaines, nous avons eu à traiter, dans le cadre de la collaboration avec nos collègues vétérinaires, 10 selles de volailles, 2 bovines et 2 murines.

Sur l’ensemble des échantillons positifs pour le *Blastocystis* sp. 50 isolats dont 39 souches humaines, 7 volailles, 2 murines et 2 bovines ont fait l’objet d’une caractérisation moléculaire.

L’ADN génomique total était extrait d’échantillons fécaux à l’aide du kit Qiagen Stool selon les recommandations du fabricant et conservé à -20 °C jusqu’à son utilisation. Chaque échantillon d’ADN a été amplifié par une PCR conventionnelle point final selon le protocole de Poirier et al. [21] qui consiste à amplifier un fragment du gène codant pour la sous-unité d’ARN 18S dont la taille est comprise entre 320 et 342 pb en utilisant les amorces sens et anti-sens suivantes:

BL 18SPPF1 sens: 5’-AGTAGTCATACGCTCGTCTCAAA- 3’ BL18SR2PP anti-sens: 5’- TCTTCGTTACCCGTTACTGC-3’

Chaque série d’échantillons incluait un témoin négatif (ADN matrice remplacée par l’eau) et un témoin positif représenté par l’ADN obtenu à partir d’une culture de *Blastocystis* ST4.

Les produits PCR, les amplicons de taille comprise entre 320 et 342 pb, après purification ont été séquencés par Chromas Pro V1.34.

Les produits de séquençage ont été comparés avec les séquences de *Blastocystis* déjà connues, disponibles auprès du Centre national d’information sur la biotechnologie (GenBank: ncbi.nlm.nih.gov).

Les ST ont été identifiés en déterminant la correspondance exacte ou la plus proche (98 % à 100 %) avec tous les ST connus de *Blastocystis.*

Les séquences obtenues dans cette étude ont été déposées dans GenBank pour attribution de numéros d’adhésion: souche murine MW332248, souches volailles MW350065 et MW391901 à MW391906, souches humaines MW377586, MW377587, MW377588 et MW391874 à MW391900.

## Résultats

Sur l’ensemble des 1 869 selles traitées sur une période de 3 années (2016-2018), 284 étaient positives soit une prévalence de 15,19 %. La prévalence de *Blastocystis* était de 7,38 %, suivie respectivement d’*Endolimax nanus* (3,79 %), *d’Entamoeba coli* (2,08 %), de *Giardia intestinalis* (1,60 %) et de *Cryptosporidium* sp. (0,21 %).

L’analyse de séquence des 50 souches n’a été contributive que pour 39 d’entre elles, dont 30 souches humaines, 7 souches de volailles, une seule murine et une seule bovine. Les séquences des 11 échantillons restants ont été difficiles à interpréter. L’analyse des séquences des souches a montré une identité de 98 à 100 % avec des séquences connues de *Blastocystis* sp.

ST3 était le ST prédominant dans notre cohorte avec 50 % des souches, suivi de ST1 avec 33,33 % et en troisième position le ST2 avec 13,33 %. Le ST4 a été identifié chez un seul patient soit 3,33 %. La seule souche bovine et la seule souche murine identifiées dans notre série se sont révélées respectivement ST6 et ST7. Cinq des 7 isolats aviaires se sont révélés ST6 (71,42 %) et les deux derniers ST7 (28,57 %).

Dans la présente étude nous avons constaté que le ST3 était plus élevé chez les sujets asymptomatiques (11/15, 73,33 %) que chez les sujets symptomatiques (4/15, 26,66 %) de même que le ST1 (7/10, 70 % asymptomatiques versus 3/10, 30 % symptomatiques). À l’inverse, le nombre de ST2 était plus élevé chez les sujets symptomatiques (3/4, 75 % versus 1/4, 25 %) (Tableau I).

**Tableau I:** Sous-type de *Blastocystis* des 30 souches algériennes et manifestations cliniques Blastocystis *subtype of the 30 Algerian strains and clinical manifestations*

Sous-type identifié	Nombre d’isolats	Statut clinique
STI	10 (33,33 %)	7 asymptomatiques: 7/10, 70 %
		3 symptomatiques: douleurs abdominales, diarrhée et prurit anal, 3/10, 30 %
ST2	4 (13,33 %)	1 asymptomatique: 1/4, 25 %
		3 symptomatiques: douleurs abdominales et diarrhée, 3/4, 75 %
ST3	15 (50 %)	11 asymptomatiques: 11/15, 73,33 %
		4 symptomatiques: douleurs abdominales et diarrhée, 4/15, 26,66 %
ST4	1 (3,33 %)	Asymptomatique

## Discussion

La prévalence de *Blastocystis* dans notre étude a été plus élevée (7,38 %) comparativement aux autres protozoaires du tube digestif. Cette prévalence est plus faible que celle rapportée lors d’une étude antérieure (11,2 %) à Oran (Ouest Algérien) par Boutellis et al. en 2021, et beaucoup plus faible que les résultats rapportés par Sebaa et al. (2018 et 2021), 32,1 % (92/287) et 33,3 % (759/2277) [6,8,27,28].

Regardés de plus près et en fonction du statut clinique, nos résultats rejoignent ceux de Sebaa et al. (2021) qui rapportent une prévalence globale de 33,33 % sur une cohorte de 2 277 prélèvements et de seulement 8 % sur 1 654 prélèvements de sujets asymptomatiques. Les mêmes auteurs rapportaient lors d’une étude précédente une prévalence globale de 32,1 % (92/287), et de 80,4 % (74/92) et 19,6 % (18/92) chez les sujets symptomatiques et asymptomatique respectivement [27, 28].

De même, Boutellis et al. (2021) trouvent sur un échantillonnage réduit une prévalence globale de 11,6 % et plus faible dans le groupe témoin (sujets asymptomatiques 8,5 %) [8]. La prévalence rapportée dans les autres pays du Maghreb était de 13 % en Tunisie sur une cohorte de 524 patients, alors qu’elle était la même que celle retrouvée dans notre étude (7,3 %) lors d’une enquête précédente et variait de 13,5 % à 15 % au Maroc sur deux études [5,14,29,34]. Toujours au Maroc, dans la région de Tétouan, cette prévalence était plus élevée (39,4 %) dans une population d’écoliers [13]. Ceci indique que la prévalence de *Blastocystis* sp. est variable d’un pays à un autre, et même d’une région à une autre au sein d’un même pays.

La faible prévalence retrouvée dans notre étude pourrait s’expliquer par plusieurs facteurs, l’examen microscopique à l’état frais est peu sensible en raison de la nature pléomorphe du *Blastocystis* qui comprend des formes granulaires, vacuolaires, kystique et amiboïdes, les formes vacuolaires et granulaires étant les plus facilement reconnues [28]. Afin de pallier ces difficultés diagnostiques, Dogrum et al. (2008) et Dagci et al. (2014) proposent la culture in vitro comme moyen plus sensible que l’examen microscopique [11, 12]. La sensibilité de l’examen microscopique est également inférieure à celle des techniques moléculaires peu utilisées dans nos pratiques courantes [5, 23]. Notre échantillonnage étant limité au centre et au Nord du pays, zone urbaine, il ne peut refléter la réalité. Pour toutes ses raisons, la prévalence de ce protiste dans notre pays pourrait être grandement sous-estimée.

La distribution des sous-types de *Blastocystis* en Algérie est assez similaire à celle observée globalement dans la plupart des pays à travers le monde, avec la prédominance de ST3, suivi de ST1 et ST2 [3]. Ces 3 ST représentent environ 96,66 % des sous-types isolés dans notre étude, rejoignant les résultats de Boutellis et al. (2021) qui confirment par la caractérisation de souches humaines (5 isolats) et de souches animales (13 isolats) de *Blastocystis* la présence des sous-types ST2 et ST3 partagés entre les deux hôtes [8].

L’absence des autres ST dans notre étude pourrait s’expliquer par le faible nombre d’isolats ou par l’échantillonnage. En effet, l’analyse de 30 isolats de patients vivant au contact d’animaux dans la région de Laghouat a révélé 6 sous-types avec la prédominance de ST1 (63,3 %) suivi de ST4 (23,3 %), ST2 et ST7 (13,3 %), ST3 (10 %) et ST5 (6,7 %) comme elle a révélé dans 30 % des cas des sous-types mixtes, ce qui est signalé également par d’autres auteurs, alors que dans notre cohorte aucune infection mixte n’a été identifiée [17,18,28,30]. Néanmoins, des disparités régionales de distribution des ST au sein de la population semblent exister [9].

Comparativement aux pays voisins, la distribution des ST en Algérie est similaire à celles de la Tunisie, de la Libye et de l’Égypte [1,3,5,17,18,30]. Cependant en Libye lors d’autres études, ST1 a montré la fréquence la plus élevée (plus de 50 % des isolats), suivi de ST3 ou de ST2 [1, 3]. En revanche, ST3 était largement prédominant dans trois études menées dans différentes régions égyptiennes [17, 18, 30].

Outre une transmission zoonotique possible de ces trois ST, leur prépondérance dans la population humaine suggère une forte transmission anthroponotique [3, 10, 32]. Toutefois dans notre étude ST1 représente 33,33 % des isolats, un sous-type trouvé principalement chez les bovidés [2], ce qui suggère qu’une proportion indéterminée d’infections humaines à ST1 dans notre série pourrait être d’origine zoonotique. La pratique d’élevage de bovin et d’ovin dans certaines régions d’Algérie pourrait être un facteur de diffusion du *Blastocystis* sp. ST1.

Paradoxalement, la seule souche bovine identifiée dans notre série a révélé un ST6, un sous-type aviaire. L’hypothèse qui pourrait expliquer ce résultat est le fait qu’en Algérie l’élevage de bovins et de volailles se fait au même endroit. Bien que chez les bovins la distribution des ST soit beaucoup plus hétérogène avec pas moins de 7 ST représentés principalement par ST1 et ST10, le ST6 a été identifié (10,53 %) en Iran sur une cohorte de 19 échantillons [3, 4, 19].

Concernant le ST4, il n’a été identifié que chez un seul patient (3,33 %) de notre cohorte, un résultat discordant de celui de Sebaa et al. (2018) (23,3 %) [28]. De même en France, Souppart et al. (2009) ont identifié le ST3 comme sous-type prédominant chez des patients infectés dans le Nord de la France alors que pour Poirier et al. (2011), le ST4 était prédominant dans une population de patients du centre de la France [23, 31]. Ces disparités peuvent refléter des sources de contamination différentes ou des variations dans la nature des populations étudiées.

Cependant nos résultats rejoignent ceux d’études réalisées en Tunisie, en Égypte et en Libye où le ST4 n’a pas encore été identifié, confirmant ainsi son absence dans les pays d’Afrique du Nord [1,5,17,18,30]. Le ST4 est un sous-type beaucoup moins fréquemment détecté ou absent en Afrique, au Moyen-Orient, en Asie et en Amérique du Nord, alors qu’il est souvent identifié en Europe [3, 23].

Ces différences suggèrent des réservoirs ou des modes de contamination différents selon les régions ou les environnements.

Dans notre cohorte, aucun ST6 à ST9 n’a été identifié parmi les isolats humains. Comparativement aux pays voisins, en Tunisie le ST7 a été identifié dans un cas de même qu’en Libye alors qu’en Égypte aussi bien le ST6 que le ST7 sont fréquemment identifiés [5, 22, 24]. Généralement hébergés par les oiseaux, les ST6 et ST7 sont appelés ST aviaires. Ils ne colonisent que rarement les mammifères y compris les humains [2, 3], suggérant leur spécificité d’hôte et le fait que les infections humaines par les ST aviaires sont d’origine zoonotique.

La caractérisation moléculaire des 7 isolats de volailles dans notre série a révélé 5 ST6 (71,42 %) et 2 ST7 (28,57 %) ce qui rejoint la littérature même si dans le cadre de notre enquête épidémiologique, le nombre d’échantillons de ces animaux soit trop limité pour permettre une comparaison avec d’autres études.

De même pour les rongeurs, une seule souche murine a été identifiée en tant que ST7 dans notre étude. Selon Cian et al. (2017) le nombre d’isolats de ces animaux sous-typés est beaucoup trop limité pour établir une quelconque tendance dans la prédominance de certains ST, même si le ST4 semble prédominant chez les rongeurs [9].

La corrélation entre statut clinique et sous-type dans notre étude a montré une prédominance de ST1 et ST3 (70 % et 73,33 %) chez les sujets asymptomatiques et inversement un nombre élevé de ST2 (75 %) chez les sujets symptomatiques. Nos résultats sont discordants avec ceux de Sebaa et al. (2018) qui ont trouvé que le ST1 était significativement plus élevé chez les patients symptomatiques (78,9 %) que les asymptomatiques (21,1 %) en accord avec Tan (2008) qui a rapporté que ST1 provenait exclusivement de patients symptomatiques contre une fraction majeure (75 % et 100 % respectivement) de ST3 chez les asymptomatiques [28, 32]. La diarrhée serait associée aux ST1, ST3 et ST4 et plus globalement chez les patients symptomatiques [1].

La pathogénie de *Blastocystis* sp. est controversée, principalement du fait que ce parasite peut être retrouvé chez des patients symptomatiques mais aussi chez des patients asymptomatiques [12, 31]. Il n’existerait à l’heure actuelle aucun consensus quant à la prédominance de certains ST plutôt que d’autres dans le développement de symptômes gastro-intestinaux ou d’une pathologie digestive précise.

La corrélation éventuelle entre ST de *Blastocystis* sp. et pouvoir pathogène du parasite a fait l’objet d’un grand nombre d’études afin de mettre en évidence cette correspondance en analysant différents types de cohortes de patients, mais les résultats restent très contradictoires.

## Conclusion

Selon cette enquête épidémiologique, le protiste *Blastocystis* sp. semble plus fréquent que le reste des protozoaires intestinaux. La caractérisation moléculaire d’isolats de ce protiste, bien que le nombre de souches soit faible, a donné une idée sur la distribution des ST en Algérie. Ainsi, 4 ST ont été identifiés parmi les souches humaines avec une prédominance de ST3, et 2 ST parmi les souches aviaires, ST6 et ST7. Ces nouveaux résultats peuvent servir à de futures études sur la diversité génétique du parasite.

La circulation des sous-types de *Blastocystis* en Algérie n’est pas encore bien déterminée. Pour cette raison, d’autres enquêtes épidémiologiques impliquant un plus grand nombre d’échantillons provenant de différents hôtes sont nécessaires pour évaluer l’épidémiologie moléculaire de ce protiste et identifier les potentiels réservoirs de l’infection humaine.

## Liens d’intérêts

Les auteurs ne déclarent aucun lien d’intérêt.

## Contribution des auteurs

S.A. Belmadani: examen parasitologique des selles et conservation des échantillons positifs.

F. Abidat, Y. Ghaffor, S. Belili: réalisation des PCR et séquençages.

S. Goura: alignement des séquences et soumission du résultat à GenBank.

F. Bachi: validation des résultats et rédaction de l’article.
